# Impact of lactisole on the time-intensity profile of selected sweeteners in dependence of the binding site

**DOI:** 10.1016/j.fochx.2022.100446

**Published:** 2022-09-16

**Authors:** Corinna M. Deck, Maik Behrens, Martin Wendelin, Jakob P. Ley, Gerhard E. Krammer, Barbara Lieder

**Affiliations:** a Christian Doppler Laboratory for Taste Research, Faculty of Chemistry, University of Vienna, Austria; b Department of Physiological Chemistry, Faculty of Chemistry, University of Vienna, Austria; c Leibniz-Institute for Food Systems Biology at the Technical University of Munich, Freising, Germany; d Symrise Distribution GmbH, Vienna, Austria; e Symrise AG, Holzminden, Germany

**Keywords:** Sweet taste receptor, Time-intensity, Cyclamate, NHDC, Aspartame, Acesulfame K

## Abstract

Currently, there is limited insight into the influence of the different binding sites of agonists and antagonists of the sweet taste receptor TAS1R2/TAS1R3 on temporal sensory properties of sweet tasting compounds. We investigated whether the binding site and a competitive or allosteric inhibition of TAS1R2/TAS1R3 influence the time-dependent sensory perception and in vitro TAS1R2/TAS1R3-activation profiles. We compared time-intensity ratings of cyclamate, NHDC, acesulfame K, and aspartame with and without lactisole with the corresponding TAS1R2/TAS1R3-activation in transfected HEK293 cells. In combination with lactisole, cyclamate and NHDC demonstrated a shift of the dose–response curve corresponding to a competitive inhibition by lactisole in the sensory and the cell experiments. Allosteric inhibition by lactisole for aspartame and acesulfame K was seen in the cell experiments, but not the sensory ratings. In conclusion, the data do not support a major impact of the binding site on the time-intensity profile of the tested sweeteners.

## Highlights

• The effect of lactisole on time-intensity profiles of selected sweeteners is shown.
• Allosteric vs. competitive inhibition of TAS1R2/TAS1R3 was used in a sensory study.
• A comparison of sensory experiments with in vitro TAS1R2/TAS1R3 activation is shown.
• Effect of lactisole on onset & lingering was not related to a specific binding site.
• The EC50 of the max. intensity correlated between *in vitro* and sensory experiments.

## 1 Introduction

The human preference for sweet taste is innate, probably because sweetness signals a caloric benefit of food (Ganchrow et al., 1983, Nelson et al., 2001, Ventura and Worobey, 2013). Beside the classical house-hold sugar sucrose, a large variety of structurally diverse compounds is known to lead to a sweet taste perception. However, the sensorial sweetness impression can differ largely between the different compounds. Especially differences in the sweet temporal profile are well-described in literature and mainly refer to differences in the onset and lingering of the sweetness (DuBois, 2016, DuBois and Prakash, 2012, Karl et al., 2020, Tan et al., 2019). The onset describes the time of appearance until the taste reaches its first maximum intensity, whereas lingering refers to the extinction time a taste remains in the mouth (DuBois et al., 1977, Karl et al., 2020). To date, the molecular basis of those differences in the temporal profile of sweet perception is not yet fully understood. In general, sweet tasting compounds are known to activate the canonical sweet taste receptor TAS1R2/TAS1R3, a class C G–protein-coupled and heterodimeric receptor. The two receptor subunits TAS1R2 and TAS1R3 are each composed of a large extracellular amino terminal domain, also known as Venus Flytrap Domain (VFD), which is linked to a Cysteine-Rich Domain (CRD) and further to a Τrans-Membrane Domain (TMD) with seven helices (Pin et al., 2003). For multiple sweet tasting compounds, at least one binding site at the TAS1R2/TAS1R3 has been identified. While sucrose and glucose can bind to the VFD of both receptor subunits, with different affinities for the two subunits (Nie et al., 2005), cyclamate and neohesperidin dihydrochalcone (NHDC) have been demonstrated to bind to the TMD of TAS1R3 only (Jiang et al., 2005, Winnig et al., 2007, Xu et al., 2004). However, the cleft formed by two lobes of the VFD at TAS1R2 subunit is regarded as the predominant binding site for many sweet tasting compounds. For example, the carbohydrate fructose, as well as the classical sweeteners sucralose, aspartame, neotame, saccharin and acesulfame K (ace K) have been shown to bind to the VFD of TAS1R2 (Masuda et al., 2012, Xu et al., 2004, Zhang et al., 2010). However, saccharin and ace K have been reported to bind with lower affinity also to the TMD of TAS1R3, which inhibits the sweet taste signaling by shifting the receptor to an inactive confirmation (Galindo-Cuspinera et al., 2006). Zhao et al. additionally proposed inhibitory residues for saccharin at the TAS1R2 by using chimeric human/mouse receptors (Zhao et al., 2022). Another compound that was previously shown to suppress the sweetness of several common sweeteners and sugars is lactisole, which binds to the TMD of the TAS1R3 subunit (Jiang et al., 2005, Jiang et al., 2005, Winnig et al., 2007, Xu et al., 2004).

Since the sweet taste receptor has multiple binding sites, different compounds can act as positive or negative allosteric modulators, or competitive inhibitors depending on the specific binding sites of the agonists and antagonists, respectively. Lactisole can thus act as an allosteric or competitive inhibitor for certain compounds based on their binding site (Servant et al., 2020, Winnig et al., 2007). Competitive inhibition by lactisole via binding to the same binding-site, for example with cyclamate and NHDC, is then characterized by a right-shift of the dose–response relationship with an enhanced EC_50_ value, but a similar E_max_ and hillslope value. In contrast, allosteric inhibition through binding to two different binding-sites, for example with aspartame and ace K, is characterized by a similar EC_50_ value in addition to a decreased E_max_ and hillslope value (May et al., 2007, Winnig et al., 2007). This concept has been successfully applied by Winnig et al. (2007), who, in addition to experiments using receptor chimera, targeted point mutations, and docking studies, used the analysis of dose–response relationships obtained from TAS1R2/TAS1R3-transfected HEK293 cells of the above–named sweeteners in combination with lactisole for the confirmation of the binding site of NHDC (Winnig et al., 2007). In addition to its sweet taste inhibiting effect, lactisole is also known to induce a delayed sweet taste, called “sweet water taste”, after rinsing with water (Galindo-Cuspinera et al., 2006).

Despite the increasing knowledge regarding the interaction of sweeteners and the TAS1R2/TAS1R3, there is only limited insight into the influence of the different binding sites of an agonist and antagonist on temporal sensory properties. Thus, we here aimed to investigate whether the binding site and a competitive or allosteric inhibition of the sweet taste receptor influence the time-dependent sensory perception and sweet taste receptor activation profiles and will translate into the corresponding changes in the time-dependent dose–response relationships. A well-established method to measure the temporal sensory properties of one specific attribute is the time-intensity (TI) measurement (Guinard et al., 1995, Ott et al., 1991). To analyze the activation of a particular GPCR as for example the sweet taste receptor, Ca^2+^-mobilization in transfected HEK293 cells is the standard method (Behrens et al., 2017, Ben Abu et al., 2021). Thus, in the present study, two sweeteners that have been proposed to bind to the TAS1R2-VFTD (ace K, aspartame) and two sweeteners that target the TAS1R3-TMD (cyclamate and NHDC) have been selected and their TI-profiles for sweet taste have been recorded in a broad range of concentrations alone or in combination with the sweet taste receptor antagonist lactisole. In parallel, dose-dependencies of the Ca^2+^-responses of TAS1R2/TAS1R3 transfected HEK293 cells were measured after stimulation with the same test compounds with or without lactisole and compared to sensory results. We hypothesized that the determined parameters for the temporal sensory properties of ace K and aspartame, will demonstrate an allosteric inhibition mode, whereas for cyclamate and NHDC, a competitive inhibition in the respective dose–response curves will be seen when applied in combination with lactisole in the sensory and cell studies.

## 2 Materials & methods

### 2.1 Materials

All compounds used for sensory evaluations were obtained in food grade (FG) quality. Citric acid, ethanol, monosodium glutamate, sodium chloride and sucrose were purchased from local supermarkets and pharmacies (Vienna, AT). Caffeine (anhydrous, 99 %, FG, W222402) and NHDC (≥  96 %, FG, W381101) was obtained from Merck KGaA (Darmstadt, DE). Acesulfame K (>  98 %), aspartame (> 99 %), cyclamate (>  99 %), lactisole (> 99 %), iron lactate-IIhydrate, and tannic acid (nat.) were kindly provided in FG quality by the Symrise AG (Holzminden, DE). Compounds used in cell experiments were aspartame, cyclamate (sodium salt, ≥ 99 %), neohesperidin dihydrochalcone (≥  95 %), acesulfame K (≥  99 %) from Sigma-Aldrich; lactisole (sodium salt, ≥ 98 %) from Cayman Chemical.

### 2.2 Sensory evaluation

For performing sensory analysis, a panel of 37 test persons (f: 26, m: 11, age: 28.6 ± 6.3 years, BMI: 22.7 ± 2.7) rated the test solutions. All panelists gave their written informed consent for participating in the panel and had to complete a sensory training. First, a training of the basic tastes sweet by 10.0 g/L sucrose, bitter by 0.3 g/L caffeine, salty by 2.0 g/L sodium chloride, sour by 0.3 g/L citric acid and umami by 0.6 g/L monosodium glutamate (DIN-EN-ISO, 2014) was conducted. For sweet and bitter thresholds ascending concentrations of sucrose and caffeine were tested (DIN-EN-ISO., 2011, Höhl and Busch-Stockfisch, 2015). Secondly, the panelists had to rank four concentrations each of a bitter, sweet (Busch-Stockfisch, 2015), metallic (iron lactate-II hydrate), and also two astringent (tannic acid, nat.), solutions in order of intensity (Karl et al., 2020). On a last training day, the evaluation on unstructured scales and the time-dependent evaluation on the computer were trained. All panelists reported to be in good general health condition, and not being under medication or pregnant. At least one hour before every training or tasting, panelists were instructed not to smoke and not to consume intense tasting food or beverage (e.g. coffee, chili, garlic, chewing gum) and to avoid in general strong hunger or fullness as well as strong odors or perfume on test days.

For evaluation of the time-dependent parameters of the selected sweet test compounds, a TI-measurement was applied, using the software EyeQuestion® 4.11.74 (Logic8 BV, NL) online. The 7–8 ascending concentrations of the test compounds were pre-tested by selected panelists (n = 3 – 4) to be in a sensory consumable range, namely 0.01 – 50 mM ace K, 0.01 – 20 mM aspartame, 0.1 – 100 mM cyclamate and 0.001 – 1.0 mM NHDC. Each test compound and concentration was tested alone and in combination with 0.46 mM (100 ppm) and 0.92 mM (200 ppm) lactisole. The concentrations were chosen because lactisole (sodium 2-(4-methoxy phenoxy) propanoate) is commonly used up to 150 ppm in food (Burdock et al., 1990) and typical cell experiments were conducted with 1.0 mM (Winnig et al., 2005). Every test solution was assessed randomly at two different test days with at least 8 panelists participating per replicate. The panelists were free to choose to participate at the different test days. A maximum of five test compounds were assessed in one session. All samples were coded with three-digit random code and presented to the panelists in randomized order. On each test day, panelists were presented five sweet solutions with 0, 25, 50, 100 and 200 g/L sucrose as a scale training for the ratings from “not at all” to “very intensive” sweetness. The panelists had to rate the intensity of the samples on an unstructured scale from “not at all” to “very intensive” sweetness for 180 seconds (*sec*) by moving the slider control in the software according to their perceived sweet intensity. The three minutes of evaluation were chosen to capture as much of the lingering as possible without wearing out the panelists during the evaluation. A total of 350 measuring time points was recorded within the pre-set time frame, especially to see differences in the first seconds of evaluation, which are important for the onset. The panelists were asked to take the sample (20 mL) into their mouth, while starting the timer and simultaneously the evaluation of sweet intensity over the time, and to spit out the sample after ten seconds while continuing the evaluation of the sweetness intensity until the end of the three minutes.

For comparison of the time-dependent properties, the following descriptors were used: the maximum intensity at the first 30 *sec* of evaluation as “max. intensity”, the time-point of the first maximum intensity as “onset” in *sec*, the intensity at 90 *sec* as “lingering” effect, and the area under the curve from the time-intensity plots as “AUC” as a marker for the overall intensity and duration.

All sensory experiments were conducted at the Christian Doppler Laboratory for Taste Research at the Institute for Physiological Chemistry, University of Vienna, Austria, at room temperature (21 – 23 °C).

Calcium mobilization assay

For the functional characterization of the human sweet taste receptor, we used HEK293 Flp-In T-Rex cells stably transfected with the G protein chimera Gα15_Gi3_, and the two subunits of the human sweet taste receptor, TAS1R2 and TAS1R3 (Galindo-Cuspinera et al., 2006). The G protein chimera and TAS1R2 subunit are constitutively expressed, whereas the expression of the TAS1R3 subunit is inducible through addition of 0.5 µg/mL tetracycline. The experiments were done exactly as before (Behrens et al., 2017, Ben Abu et al., 2021). Briefly, cells were seeded into 96-well plates (black, flat clear bottom) treated with 10 µg/mL poly-d-lysine and grown in low-glucose DMEM supplemented with 10 % fetal bovine serum, 100 U Penicillin/mL, 0.1 mg/mL Streptomycin, 2 mM l-glutamine, at 37 °C, 5 % CO_2_, saturated air-humidity overnight. About 24 h before the experiment tetracycline was added to induce TAS1R3 expression. Next, cells were loaded for 1 h with Fluo-4 am in the presence of 2.5 mM probenecid and washed twice with C1-buffer (130 mM NaCl, 5 mM KCl, 10 mM HEPES, 1 mM sodium pyruvate, and 2 mM CaCl_2_, pH 7.4). Then, plates were placed in a fluorometric imaging plate reader (FLIPR^tetra^, Molecular Devices, San Jose, CA, United States) for automated compound application and measurement of fluorescence changes. Fluorescence changes of control wells not induced with tetracycline were subtracted from the data. Next, measurements were corrected for background fluorescence. Dose-response relationships of three independent experiments each performed in duplicates were calculated with SigmaPlot 14.0 software using the function f(x) = min+(max − min)/(1+(x/EC_50_)^nH^).

### 2.4 Computational and statistical analysis

TI-ratings were recorded and analyzed using the software EyeQuestion® 4.11.74 (Logic8 BV, NL) online and MS Excel 16.0 (Microsoft Corporation, USA). The statistical analysis and graphical representation were performed with GraphPad Prism 9.0 for sensory results and with SigmaPlot 14.0 for cell experiments. To evaluate the sensory dose–response effects of ascending sweetener concentration w/o lactisole, an asymmetrical five-parameter curve (Richard́s five-parameter dose–response curve) was fitted. The data are presented as means ± SEM from at least 16 single evaluations. Statistical significance between the different test compounds and concentration-dependent effects was assessed by two-way ANOVAs with Tukey post hoc test using Graph Pad Prism. The normal distribution of the data sets was checked by evaluation of the kurtosis (between −7 to + 7) and the skewness (between −3 to + 3). Equal variance was tested using Levenés test. In case the assumptions of a normal distribution and equal variance were not met, the non-parametric ANOVA on ranks (Kruskal-Wallis Test) with Dunn's post-hoc test without the calculation of interaction was applied. A Pearson product moment correlation was conducted to investigate relationships between sensory results and cell responses of the tested sweeteners.

## 3 Results

In the present study, the impact of selected sweet tasting compounds, namely acesulfame K (ace K), aspartame, cyclamate and NHDC on the sweet taste receptor TAS1R2/TAS1R3 was evaluated. First, TI-measurements for sweetness of the test compounds were conducted in order to obtain time-dependent parameters in dependence of concentration with and without the combined application of the sweet taste inhibitor lactisole. As lactisole acts either as a competitive or allosteric inhibitor for sweeteners based on their binding site at the sweet taste receptor, the aim of this experiment was to detect whether a different binding site at the sweet taste receptor also translates into differences on the temporal sensory profile.

**Fig. 1:**
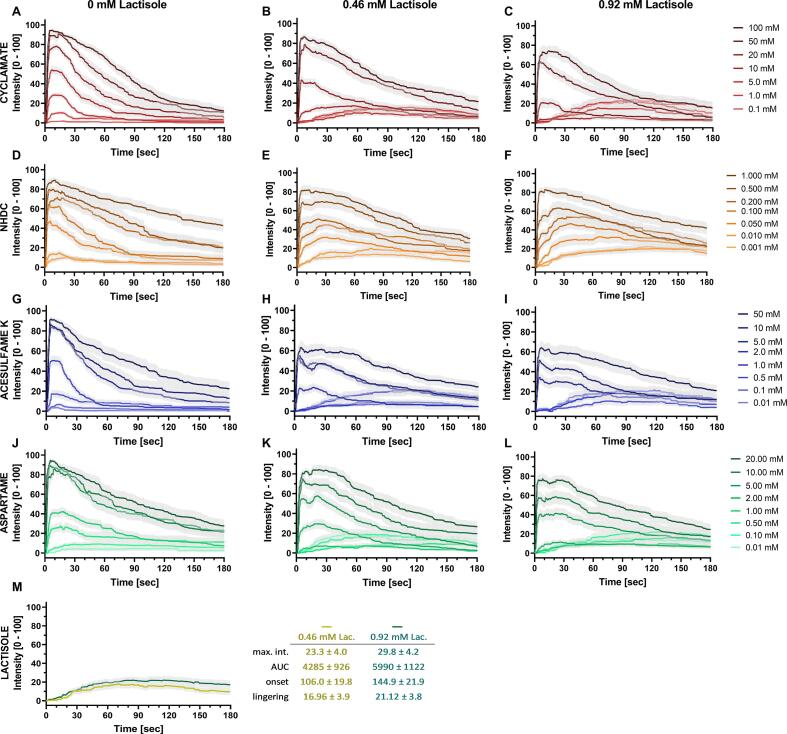
Time-intensity curves of 0.01 – 50 mM cyclamate (A-C), 0.001 – 1.0 mM neohesperidin dihydrochalcone (NHDC) (D-F), 0.01 – 50 mM acesulfame K (G-I), 0.01 – 20 mM aspartame (J-L), each without and in combination with 0.46 mM and 0.92 mM. Time-intensity curves of 0.46 mM and 0.92 mM lactisole (Lac.) separately and the temporal markers maximum intensity (max.int.), AUC, onset and lingering (M). 2 rep. with n = 18 – 27 single evaluations, presented as mean ± SEM.

A broad, for panelists acceptable concentration range of each test compound was used, in absence or presence of 0.46 mM and 0.92 mM lactisole (100 ppm and 200 ppm). Fig. 1 shows the time-intensity profiles of the tested concentrations of cyclamate (A-C), NHDC (D-F), ace K (G-I) and aspartame (J-L) w/o lactisole. Higher concentrations of the sweeteners led to a higher intensity curve of all test compounds with more pronounced lingering (two-way-ANOVA, p < 0.0001), whereas the lowest test concentrations were hardly perceived as sweet. Cyclamate (Fig. 1 A-C) showed a strong dose-dependency of the maximum intensity in the time-intensity curves without major discontinuities in the perceived intensity in the tested concentrations. The inhibition of cyclamate’s sweetness by lactisole was effective for all concentrations and dose-dependent with effective inhibition for up to 5.0 mM and 10.0 mM cyclamate for 0.46 mM and 0.92 mM of lactisole (Fig. 1 B&C). Lactisole had a dose-dependent effect on the sweetness of NHDC (two-way-ANOVA, p < 0.001). A proper inhibition was detected up to 0.2 mM NHDC with 0.46 mM lactisole (Fig. 1 E) and up to 0.5 mM NHDC with 0.92 mM lactisole (Fig. 1 F). In case of ace K and aspartame, a saturation of the perceived maximum intensity was present for the three highest concentrations. Interestingly, the intensity of ace K (Fig. 1 G-I) and aspartame (Fig. 1 J-L) rose sharply by increasing the concentration from 2 mM to 5 mM, regardless of whether lactisole was added or not. The addition of 0.46 mM and 0.92 mM lactisole to up to 1 mM ace K effectively inhibited the sweetness over the time (Fig. 1 H & I). Aspartame revealed similar TI-curves with a strong lingering effect in all test concentrations compared to ace K. The addition of 0.46 mM lactisole (Fig. 1 K) inhibited the sweetness of aspartame up to a concentration of 1.0 mM of aspartame, and up to 2.0 mM when 0.92 mM of lactisole was used (Fig. 1 L).

Notably, an increased sweetness in the combinations with lactisole was reported by the panelists after around 30 *sec*, which is similar to the temporal sweetness recordings of lactisole alone (Fig. 1 M). This increased sweetness after approximately 30 *sec* was seen for all combinations at which lactisole was able to completely inhibit the sweetness of the test compounds and was higher for 0.92 mM lactisole compared to 0.46 mM lactisole.

**Fig. 2:**
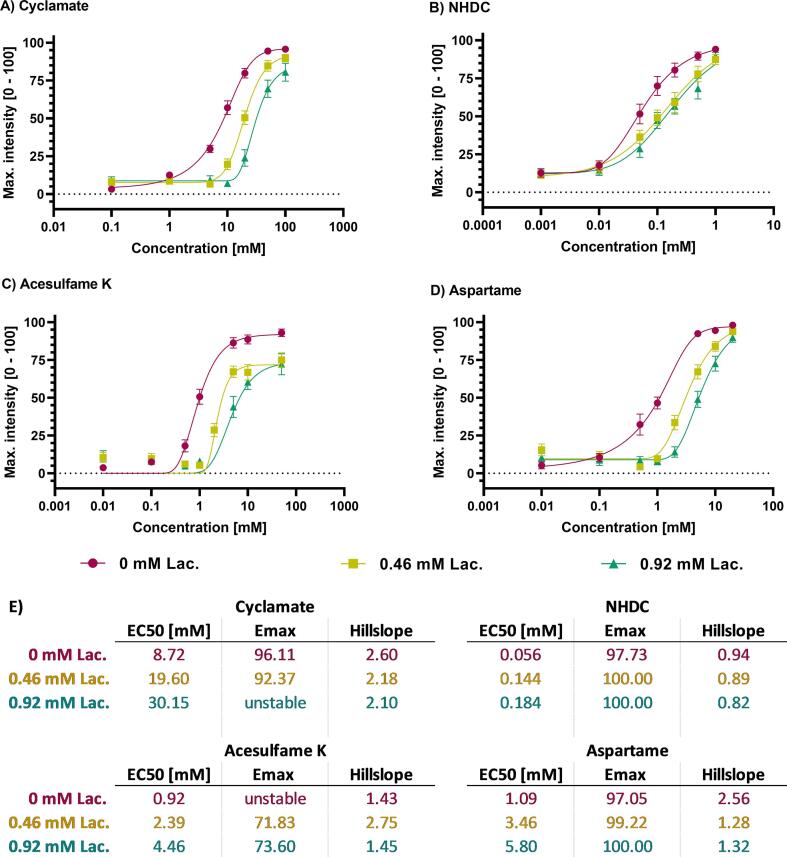
Max. intensity [0 – 100 s] of (A) cyclamate (0.1 – 100 mM), (B) NHDC (0.001 – 1.0 mM), (C) acesulfame K (0.01 – 50 mM) and (D) aspartame (0.01 – 20 mM); presented as mean ± SEM; 2 rep. with n = 18 – 27 single evaluations for combination with 0 mM, 0.46 mM and 0.92 mM lactisole (Lac.); (E) EC50 [mM], Emax and hillslope calculated with GraphPad Prism 9 curve fitting with an asymmetric five parameter curve, top < 100 and bottom > 0.

Next, the effect of lactisole in dependence of the different binding sites of the sweeteners on the temporal markers maximum intensity, AUC, onset, and lingering was analyzed. In more detail, we hypothesized that the dose–response relationships for the selected markers will show a competitive inhibition in combination with lactisole for cyclamate and NHDC, and an allosteric inhibition in combination with ace K and aspartame. First, the max. intensity was plotted against all tested concentrations. The dose–response curves for ace K, aspartame, NHDC and cyclamate obtained from the maximum intensities of the respective time-intensity curves showed a sigmoidal pattern and are displayed in Fig. 2. The applied two-way ANOVA models revealed that the inhibitory effect of lactisole was dependent on the sweetener concentrations, except for NHDC.

For cyclamate, the applied model gave similar E_max_ values without or with 0.46 mM lactisole, and due to the higher variation, no E_max_ could be calculated for 0.92 mM lactisole. The corresponding EC_50_ values for cyclamate increased with higher lactisole concentration. The hillslope remained at a similar level for the effects with or without lactisole, see Fig. 2 A. The model calculated based on the max. intensities of NHDC showed that the E_max_ values remained similar with increasing lactisole concentrations (Fig. 2 B), and the calculated EC_50_ values for NHDC rose from 0.056 mM without lactisole to 0.14 mM with 0.46 mM lactisole, and 0.18 mM with 0.92 mM lactisole. Similar to cyclamate, the hillslopes of the NHDC dose–response curves remained similar (Fig. 2 B, p > 0.05). To summarize, the curve shifts for cyclamate and NHDC mostly follow the expectation for a competitive binding mode with lactisole. For ace K, the E_max_ was reduced by 19.34 % and 22.38 % (p < 0.01), respectively, for 0.46 and 0.92 mM lactisole (Fig. 2 C). The EC_50_ values of ace K curves increased with increased lactisole concentrations without dose-dependent changes in the hillslopes (Fig. 2 C). In case of aspartame (Fig. 2 D) the EC_50_ values increased with increasing lactisole concentrations, but the hillslope of the aspartame dose–response curves decreased from 2.56 without lactisole to 1.28 at 0.46 mM and 1.32 with 0.92 mM lactisole. Thus, the curve shift for ace K mainly followed the expected allosteric inhibition mode with lactisole, which was not consistently the case with aspartame.

**Fig. 3:**
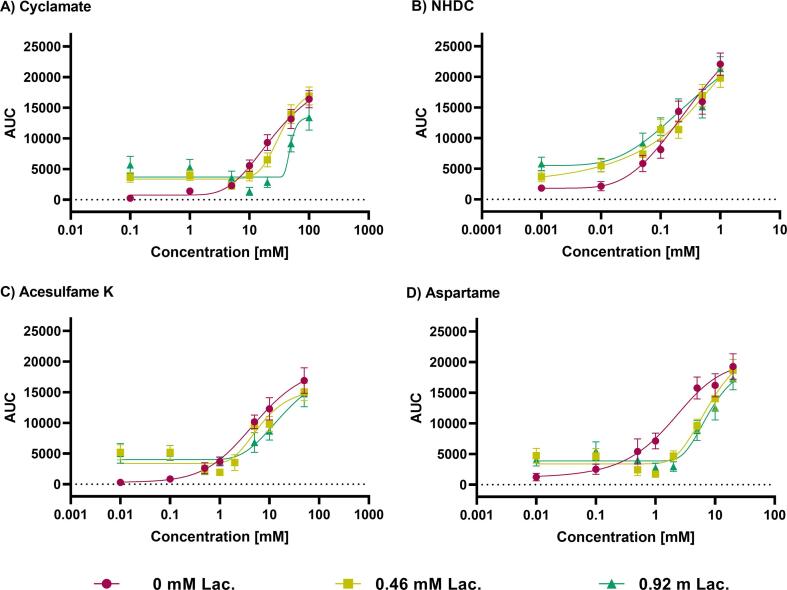
Area under the curve (AUC) of time-intensity curves of (A) cyclamate (0.1 – 100 mM), (B) NHDC (0.001 – 1.0 mM), (C) acesulfame K (0.01 – 50 mM) and (D) aspartame (0.01 – 20 mM); presented as mean ± SEM; 2 rep. with n = 18 – 27 single evaluations for combination with 0 mM, 0.46 mM and 0.92 mM lactisole (Lac.).

As a second parameter, the AUC of the TI-curves in the tested range of concentrations was compared, to assess the summated overall sweetness impression exerted by the different sweeteners w/o lactisole as a function of duration and intensity. The concentration dependency of the AUC for the four sweeteners w/o lactisole is displayed in Fig. 3. In contrast to the dose-dependent curves of the max. intensities, no saturation for the AUC was reached with the highest concentrations of sweeteners, although a dose-dependency was seen for the sweeteners without lactisole (Fig. 3, red lines). Similar to the max. intensity, the AUC of the time-intensity curves showed that the effect of lactisole was dependent on the sweetener concentration, demonstrated by the significant interaction of the sweetener concentration and the inhibitory effect of lactisole in the two-way ANOVA (p < 0.05), except for NHDC. In lower concentrations of the sweeteners, the sweet aftertaste of lactisole after 30 *sec*, as described above, led to increased AUCs. Similar E_max_ values were obtained for the combination with 0.46 mM or 0.92 mM lactisole, although in the combinations with lactisole, the rise of the curves was present only from a concentration of 10 mM or 20 mM cyclamate respectively (right shift).

In addition, the onset, the time until the max. intensity is first reached, (see Supplemental Fig. S1) and the lingering, analyzed as intensity at t = 90 *sec* (Supplemental Fig. S2) were evaluated for a dose-dependent effect. Regarding the onset (Supplemental Fig. S1), the sweet aftertaste of lactisole after 30 *sec* in the lower sweetener concentration dominated and overruled the intrinsic sweetness of the test compounds, as a sweet perception for the sweet taste inhibitor lactisole was reported by the panelists after around 30 *sec*. This can be seen from the dose–response plots of the onset, at which the onset was largely increased within the lower test concentrations at which the sweeteners did not show significant sweetness on their own. There was no clear difference between the test compounds based on their binding site detectable. Similarly, there were no major differences in the dose–response curves of the different sweeteners for the lingering (Supplemental Fig. S2), however, all compounds showed an increased lingering with higher concentration of the test compound (sweetener concentration effect p < 0.0001) and only for cyclamate the effect of lactisole was dependent on its concentration (p < 0.001). Furthermore, similarly to the onset, there was an increased lingering for all tested sweeteners in combination with lactisole which was independent of the binding site.

**Fig. 4:**
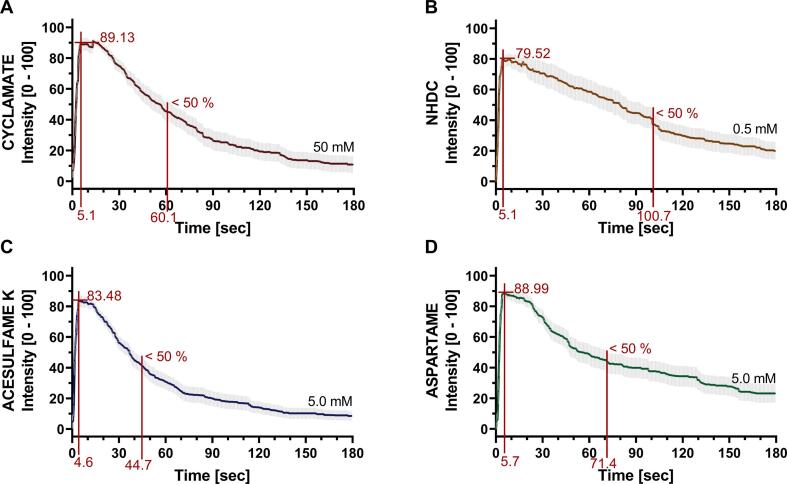
Time-intensity curves of (A) 50 mM cyclamate, (B) 0.5 mM NHDC, (C) 5.0 mM acesulfame K, and (D) 5.0 mM aspartame. All curves are marked in red for maximum sweetness, half maximum intensity and their temporal occurrences (onset and lingering time). Data presented as mean ± SEM; 2 rep. with n = 19 – 24 single evaluations.

The onset and lingering were additionally analyzed by comparing the first onset and decay time for a concentration at the sweet intensity saturation level (Fig. 4). The onset was in a similar range for all test compounds, namely 4.6 *sec* for ace K, followed by cyclamate and NHDC with 5.1 *sec* and aspartame with 5.7 *sec*. The lingering effect was analyzed by comparing the time point at which a sweet compound reached < 50 % of its maximum sweetness. The fastest sweetness decay was recorded for ace K with < 50 % of maximum intensity after 44.7 *sec*, followed by cyclamate with 60.1 *sec* and aspartame with 71.4 *sec*. NHDC showed the most prolonged lingering aftertaste with 100.7 *sec*, see Fig. 4. In summary, the temporal parameters were not consistently different between the orthosteric (TAS1R2-VFD) or allosteric (TAS1R3-TMD) binding site.

**Fig. 5:**
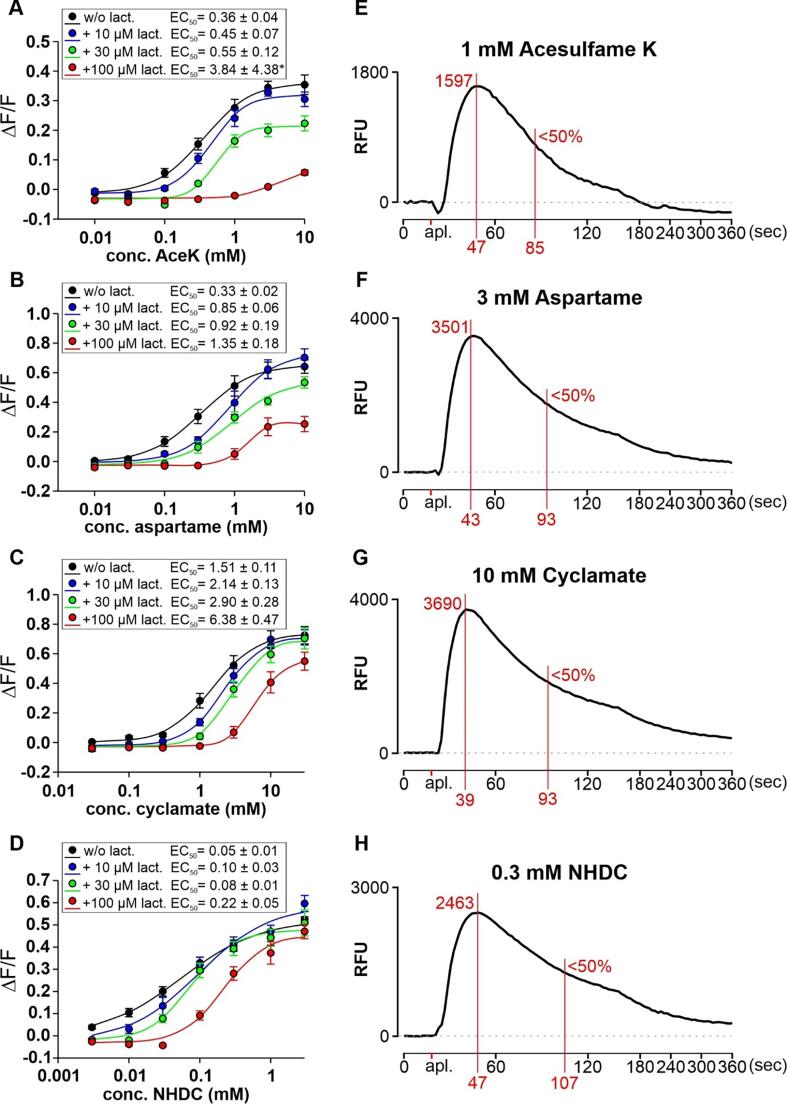
*In vitro* analyses of cells expressing the human sweet taste receptor. Left panels (A-D): Dose-response relationships of HEK293 Flp-In *T*-Rex-Gα15Gi3-TAS1R2/TAS1R3 cells stimulated with increasing concentrations of the sweeteners acesulfame K (Ace K) (A), aspartame (B), cyclamate (C), and neohesperidin dihydrochalcone (NHDC) (D) in the presence or absence of the sweet taste inhibitor lactisole (lact.). The agonist concentrations in millimolar (mM) are labeled on the logarithmically scaled x-axes, the relative fluorescence changes (ΔF/F) on the y-axes. The curves are color coded according to the fixed lactisole concentrations indicated in the insets. Determined EC50 concentrations are provided in the insets. Right panels (E-H): Raw traces of fluorescence changes of cells expressing TAS1R2/TAS1R3 at selected concentrations of sweeteners (E, Ace K; F, aspartame; G, cyclamate; H, NHDC). The traces of 3 independent experiments performed in duplicates were averaged and thus, reflect 6 wells receiving the identical treatment. The fluorescence changes (RFU) are plotted on the y-axes and the measurement time in seconds (*sec*) is shown on the x-axes. The times until reaching the peak amplitudes (including 20 *sec* of baseline monitoring prior to agonist application) as well as the corresponding peak amplitudes are indicated with red lines and printing. As only one trace (Ace K) crossed the baseline upon prolonged monitoring, the time points where the traces fell below 50 % of the respective peak fluorescence are indicated with red lines and printing.

The results of the sensorial time-intensity measurements were compared to activation of the sweet taste receptor in transfected HEK293 cells. The functional analyses of a sweet taste receptor expressing mammalian cell line confirmed previous results on the differential lactisole sensitivities of the sweet taste receptor responses to sweeteners (Winnig et al., 2007). Sweeteners binding at the VFD of the TAS1R2 subunit showed pronounced depressions of their maximal signal amplitudes at elevated lactisole concentrations (Fig. 5, ace K (A) and aspartame (B)). Sweeteners binding to the TMD of the TAS1R3 subunit overlapping with the binding site for lactisole exhibited mostly a right shift in the dose–response relationships without comparable pronounced depressions of the maximum signal amplitudes (Fig. 5, cyclamate (C) and NHDC (D)). The exemplarily fluorescence traces shown in Fig. 5, (E-F) demonstrated that the onset and the decay of sweet taste receptor signal deviate among different sweeteners also *in vitro*. Whereas sweet taste receptor expressing cells stimulated with 10 mM of cyclamate (Fig. 5 G) reaches peak activity already after 39 *sec* (19 *sec* after agonist application at 20 *sec*), Ace K (Fig. 5 E) and NHDC (Fig. 5 H) stimulated cells required 47 *sec* (27 *sec*). Aspartame responses (Fig. 5 F) with 43 *sec* (23 *sec*) fell between these extremes. Thus, the activation of the sweet taste receptor via the orthosteric (TAS1R2-VFD) or allosteric (TAS1R3-TMD) binding site seems to have no consistent effect on the speed of signal onset. Also signal decays showed considerable differences. The only sweetener-induced receptor activation showing a signal decay back to baseline or even below was documented for Ace K (Fig. 5 E) after about 3 min, whereas aspartame (Fig. 5 F), cyclamate (Fig. 5 G) and NHDC (Fig. 5 H) stimulated cells maintained signals above the initial baseline for 6 min and beyond. Therefore, the times where signals decreased just below 50 % of the maximum signal amplitudes were monitored as well. Here, the fastest signal decrease was seen for Ace K (85 *sec*, 65 *sec* after agonist application at 20 *sec*) and the slowest for NHDC (107 *sec* and 87 *sec*, respectively). Aspartame and cyclamate exhibited identical signal decay times with 93 *sec* (73 *sec*). Again, signal decay seems to be independent on the interaction site.

**Fig. 6:**
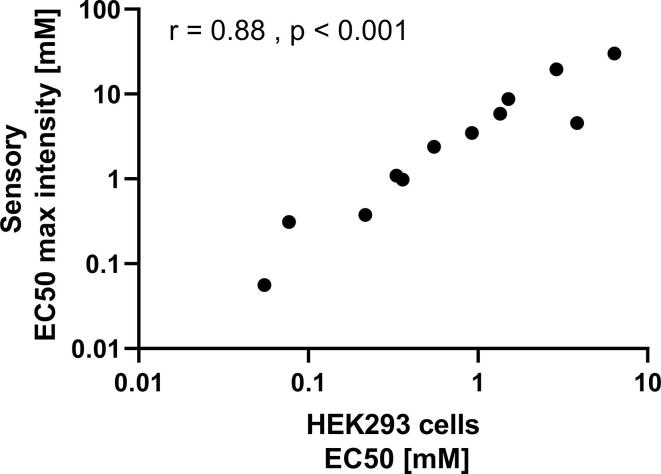
Comparison of EC50 values calculated from sensory time-intensity (TI) curves n ≥ 16) and transfected HEK293 cells (n ≥ 3) for NHDC, cyclamate, acesulfame K and aspartame w/o lactisole (sensory 0.46 and 0.92 mM, cells 30 and 100 µM). Statistics: Pearson product moment correlation.

A direct comparison of the obtained EC_50_ values for the sensory data and derived from the transfected HEK293 cells revealed a strong correlation (Fig. 6), with r = 0.88 (p < 0.001) for the EC_50_ of max. intensity of sensory to EC_50_ of response of HEK296 cells.

## 4 Discussion

The present study examined the impact of the binding site of the high-impact sweeteners ace K, aspartame, cyclamate, and NHDC when applied in combination with the sweet taste inhibitor lactisole on TI sweetness profiles in order to investigate a potential connection between the binding site and the impact on the temporal profile. We hypothesized that lactisole, which binds to the TMD of the TAS1R3 subunit of the sweet taste receptor, will lead to either allosteric (ace K, aspartame) or competitive (cyclamate, NHDC) inhibition with typical shift in the dose–response relationships.

As markers for the temporal characteristics of the sweetness perception, the maximum intensity, the AUC from the respective TI-plots, as well as onset and lingering were investigated. As expected, with rising concentration of the sweeteners, also the sweetness rating, represented by the maximum intensities as well as the AUC of the corresponding TI- plots were increased. Two, respectively three for the cell experiments, different concentrations of lactisole were included in the present study which are in a relevant range for food applications and cell experiments (Burdock et al., 1990, Winnig et al., 2007). A sweet perception for the sweet taste inhibitor lactisole was reported by the panelists after around 30 *sec*. This sweet aftertaste of lactisole was dose-dependent and also present at lower sweetener concentrations at which lactisole inhibited the sweetness of the test compounds completely. A certain sweet aftertaste of lactisole and other sweet taste inhibiting compounds is well-known and was previously described as “sweet water taste”, as it can be induced by rinsing with water after the inhibitor stimulus (DuBois, 2016, Galindo-Cuspinera et al., 2006, Sigoillot et al., 2012). To elucidate the molecular bases of this effect, Galindo-Cuspinera et al. (2006) investigated the signal of TAS1R2/TAS1R3 transfected HEK293 cells and as well sensory approaches after stimulation with several sweeteners and sweet inhibitory compounds before and after rinsing with water. They concluded from their experiments that the sweet water effect is based on a lactisole-induced shift of the TAS1R2/TAS1R3 to its inactive conformation. Rinsing with water then dissociates lactisole from the receptor and changes the balance from the inhibited receptor state towards a constitutively active state, inducing stimulus transmission with the following sweet impression. Furthermore, they suggest the sweet water effect as an identifying feature for sweet taste inhibitors (Galindo-Cuspinera et al., 2006). The results of the present study lead to the assumption that after approximately 30 *sec*, also saliva secretion, without the necessity of rinsing with water, can induce a sweet aftertaste impression, which supports the idea of the dissociation from the receptor to be important for the sweet water taste of lactisole. We propose that not only water can induce the sweet lactisole effect, but also salivary secretion during tasting. Because of the clear noticeable sweet-water effect of lactisole, we only used the values up to 30 *sec* to identify the maximum sweetness for the sweeteners.

Focusing on the TI-parameters and the effect of lactisole on the different sweeteners, the shift of the dose–response relationships of the maximum intensity for cyclamate and NHDC fit into the model of competitive inhibition in combination with lactisole. Similarly, the shifts in the dose–response relationships of the AUC can be best explained by a competitive inhibition model. As the binding site of cyclamate, NHDC, and lactisole has been previously shown to be located at the TMD of TAS1R3 (Jiang et al., 2005, Jiang et al., 2005, Winnig et al., 2007, Xu et al., 2004), these results are as expected and are consistent with the results by Winnig et al. obtained with receptor cell models *in vitro* (Winnig et al., 2007) and additionally confirmed by the here presented cell experiments. Nevertheless, for all analyzed sweet sensory parameters, the concentrations of NHDC did not influence the effect of lactisole, as it was seen for the other sweeteners by interaction of the two-way-ANOVA factors. A reason for these sensory differences and independency of lactisole could be the high affinity of NHDC for the sweet taste receptor, which is reflected by a sensory sweetness factor of approximately 900 compared to around 30 for cyclamate, both compared to 5 % sucrose (Schiffman et al., 1995). This high potency of NHDC suggests a strong affinity of NHDC for its binding site. An effective displacement of lactisole even at low concentrations of NHDC is consequently observed, since the binding site of NHDC and lactisole are overlapping.

The inhibitory effect of lactisole on ace K and aspartame was expected to reflect an allosteric inhibition mode, as these two sweeteners have been described to bind with high affinity to the VFD of TAS1R2 (Maillet et al., 2015, Masuda et al., 2012). The dose–response relationships of the maximum intensities of aspartame are similar to the cell-based single receptor assay, however, the E_max_ in the sensory experiments was less reduced than expected. This was not the case in the in vitro experiments, where pronounced reductions in the maximal signal amplitudes already at 100 µM of lactisole were evident. A reason for the discrepancy between the results obtained from the cell model and the sensory study regarding the E_max_ might be that some of the test compounds such as ace K are also activating other taste receptors, e.g. bitter taste receptors (Kuhn et al., 2004), that might interfere with the reporting of the sweet perception in the sensory studies. However, the present study focused on sweetness only, leaving out attributes such as bitterness, astringency or metallic impressions. In addition, the negatively charged lactisole could exhibit a lower bioavailability due to its interaction with basic and proline-rich salivary proteins. This concept has been previously suggested by Canon et al. for tannins and polyphenols (Canon et al., 2021, Canon et al., 2013). The interaction of lactisole with salivary proteins would thus lead to a higher required amount of lactisole when used in oral application. As a further limitation it must be noted that in the cell-based assay of the present work, sweeteners with or without lactisole remained in the wells, while the sensory experiments were done in sip-and-spit mode. In addition, the cell assay does not mimic increasing dilution by salivary flow. This could influence especially the lingering due to a longer presence of the compounds at the receptor sites in the cell experiments. Thus, one would have to assume that part of the signal decay in vivo is due to dilution and not to receptor desensitization. Accordingly, it can be concluded that the in vitro experiments might tend to underestimate while the in vivo experiments tend to overestimate the receptor kinetics. An even more realistic picture of the in vitro receptor kinetics might be obtained by using a superfusion approach to dynamically modulate stimulus concentrations and hence, mimic saliva dilution and swallowing better.

For ace K, a significant reduction of the E_max_ was shown, although the increased EC_50_ values in combination with lactisole do not fit into an allosteric inhibition model. For typical allosteric inhibition induced by lactisole, a similar EC_50_ and reduced hillslope would have been expected (Bindslev, 2013, Winnig et al., 2007) and was also shown by the here presented cell results. However, not only simple allosteric or competitive inhibition is possible, but as well mixed effects, such as allo-mixed-competitive inhibition (Bindslev, 2013), which could be also suitable for ace K and aspartame here. No such effect that would argue for a second binding site to fit in a two-state allosteric model was shown for aspartame in this study (Galindo-Cuspinera et al., 2006), although it has to be noted that aspartame was tested in lower concentrations only that may not have been high enough to detect a low-affinity binding site. Looking at the TI-curves of ace K and aspartame, a steep increase in the mean maximum sweet intensity was recorded between 2 mM and 5 mM for aspartame, and 0.5 mM and 1 mM for ace K. Beside the binding site, also specific binding residues may play a role for further taste transduction. For example, Masuda et al. (2012) suggested by using a combined approach of molecular modelling, concentration-dependent Ca^2+^-release of heterologous transfected cells and point mutations, that the binding sites for ace K and aspartame, although located both at the VFD of TAS1R2, have strikingly different and specific binding residues (Masuda et al., 2012). Since the results of the present sensory study reflect the results of the single-receptor model very well, we exclude the involvement of an unknown alternative sweet-signaling pathway for the here tested compounds cyclamate, NHDC, aspartame and ace K. The present study focused on the time-dependent attributes onset and lingering for the four test compounds with or without lactisole addition as well. However, a relationship to the binding site could not be concluded for all four sweet tasting compounds based on the present data. This suggests that the binding site does not play a major role for the temporal profile or that the effect is overruled by other taste signals that occur simultaneously at oral applications. More compounds would be needed in following studies to confirm this effect. There are some hypotheses about the origin of onset and lingering. Schiffman et al. (2007) hypothesized that the size and complexity of a molecule impact onset and lingering either by reducing the diffusion to the receptor or by enhanced time to orient properly to the receptor or by the demand of a multistep progress of several binding interactions (Schiffman et al., 2007). Supporting this hypothesis, Karl et al. (2020) demonstrated that an increased onset is primarily related to an increasing number of aromatic rings, double bonds, ketones and higher MlogP (Karl et al., 2020), indicating more complex structures. Furthermore, the study showed that an increased lingering, together with sweetness rating, is related to the physicochemical descriptors molecular weight [g/mol], complexity, heavy atom count, rotatable bonds, C-atoms, bound glucose, glycone length, area polar surface [A2], defined atom stereocenter count, acceptors, donors, and OH-groups (Karl et al., 2020). Also, an earlier hypothesis described by DuBois (2011) suggested that large molecules such as rebaudioside A will non-specifically bind to cell membranes in the oral cavity, leading to a long-lasting lingering effect by enabling re-binding to the receptor (DuBois, 2011). The recorded onset values were all in a similar range in the sensory recordings, the cell experiments showed a greater variance with a more pronounced delay in onset of ace K and NHDC at the cell experiments. In addition, the kinetic responses obtained from the single receptor cell model in the present study show that decay of the fluorescent signal resembles the lingering in the sensory study. It can be concluded that the onset and lingering directly depend on the activation of TAS1R2/TAS1R3, which supports previous assumptions by DuBois and colleagues (DuBois, 2011). However, the results of the present study thus exclude a role for a mucus membrane which is lacking in the cell model. Tan et al. (2019) summarized that a prolonged sweet intensity is induced by higher affinities of non-nutritive sweeteners to the binding site of taste receptor (Tan et al., 2019). However, to date, a specific binding-site was not shown to be important for a prolonged onset and lingering of sweet compounds. Also in the present study, the effect of lactisole on the onset and lingering time of the test compounds was not associated with a specific binding site and does not support the idea that the binding site is the major driving force for the temporal sensory profile of sweeteners. However, the detailed mechanism leading to an extended or shortened onset and lingering remains unknown and further research is required.

To summarize, in combination with lactisole, cyclamate and NHDC demonstrate a shift of the dose–response curve corresponding to a competitive inhibition by lactisole in the sensory and the single receptor cell experiments. In contrast to the expectations, aspartame was able to overrule the lactisole inhibition in higher concentrations in the sensory experiments, which could argue for a second, low-affinity binding site at the TAS1R3-TMD. Moreover, the effect of lactisole on the temporal markers of the sensory profile AUC, onset, and lingering for the sweeteners was independent of the major binding site of the sweeteners. In conclusion, the data do not support a major impact of the binding site on the time-intensity profile of the tested sweeteners. Future studies are needed to assess the effect of lactisole and further compounds to confirm their intensity and lingering effects in cell experiments related to sensory properties.

## CRediT authorship contribution statement

**Corinna M. Deck:** Conceptualization, Methodology, Software, Validation, Formal analysis, Investigation, Data curation, Writing – original draft, Writing – review & editing, Visualization. **Maik Behrens:** Conceptualization, Methodology, Software, Validation, Formal analysis, Investigation, Resources, Data curation, Writing – original draft, Writing – review & editing, Visualization. **Martin Wendelin:** Conceptualization, Methodology, Writing – review & editing. **Jakob P. Ley:** Conceptualization, Methodology, Resources, Writing – review & editing, Supervision, Project administration, Funding acquisition. **Gerhard E. Krammer:** Conceptualization, Resources, Writing – review & editing, Supervision, Funding acquisition. **Barbara Lieder:** Conceptualization, Methodology, Validation, Formal analysis, Investigation, Resources, Data curation, Writing – original draft, Writing – review & editing, Visualization, Supervision, Project administration, Funding acquisition.

## Declaration of Competing Interest

The authors declare the following financial interests/personal relationships which may be considered as potential competing interests: The authors M. Wendelin, J. P. Ley, and G. E. Krammer are employees of the Symrise Distribution GmbH or Symrise AG, respectively.
