# Supplementary material for: Impact of lactisole on the time-intensity profile of selected sweeteners in dependence of the binding site
Source: Food Chem X. 2022 Sep 16;15:100446. doi: 10.1016/j.fochx.2022.100446 (PMC9532755; doi:10.1016/j.fochx.2022.100446)
Supplement: Supplementary data 1 [file mmc1.doc]

**Impact of lactisole on the time-intensity profile of selected sweeteners in dependence of the binding site**

Corinna M. Deck ^1,2^, Maik Behrens ^3^, Martin Wendelin ^4^, Jakob P. Ley ^5^, Gerhard E. Krammer^5^, Barbara Lieder ^1,2^,*

^1^ Christian Doppler Laboratory for Taste Research, Faculty of Chemistry, University of Vienna, Austria
^2^ Department of Physiological Chemistry, Faculty of Chemistry, University of Vienna, Austria
^3^ Leibniz-Institute for Food Systems Biology at the Technical University of Munich, Freising, Germany
^4^ Symrise Distribution GmbH, Vienna, Austria
^5^ Symrise AG, Holzminden, Germany

**Supplemental Material**


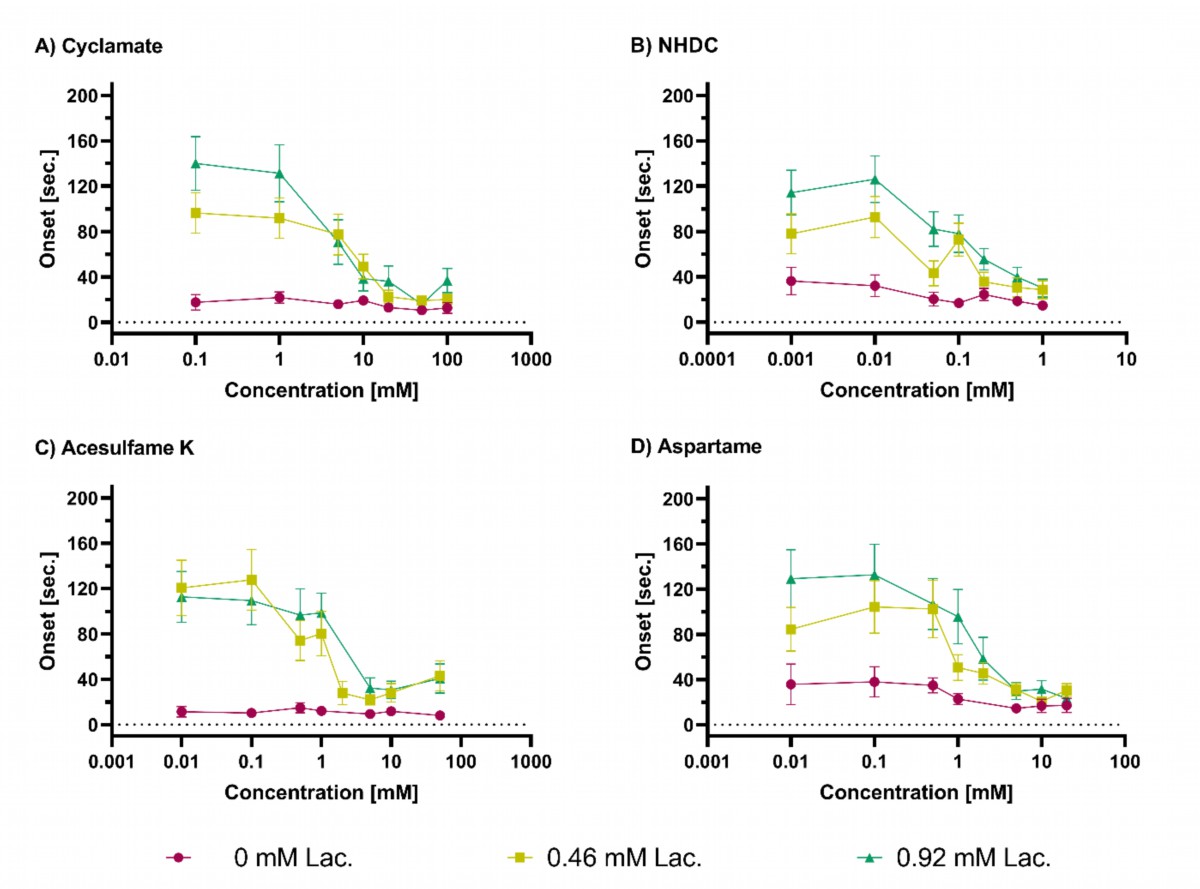


Supplemental Fig. S1: Onset, time until first maximum reached [sec.] of cyclamate (0.1 ‑ 100mM), NHDC (0.001 ‑ 1.0mM), acesulfame K (0.01 ‑ 50mM) and aspartame (0.01 ‑ 20mM); presented as mean ± SEM; 2 rep. with n= 18 ‑ 27 single evaluations for combination with 0 mM, 0.46 mM and 0.92 mM lactisole (Lac.).


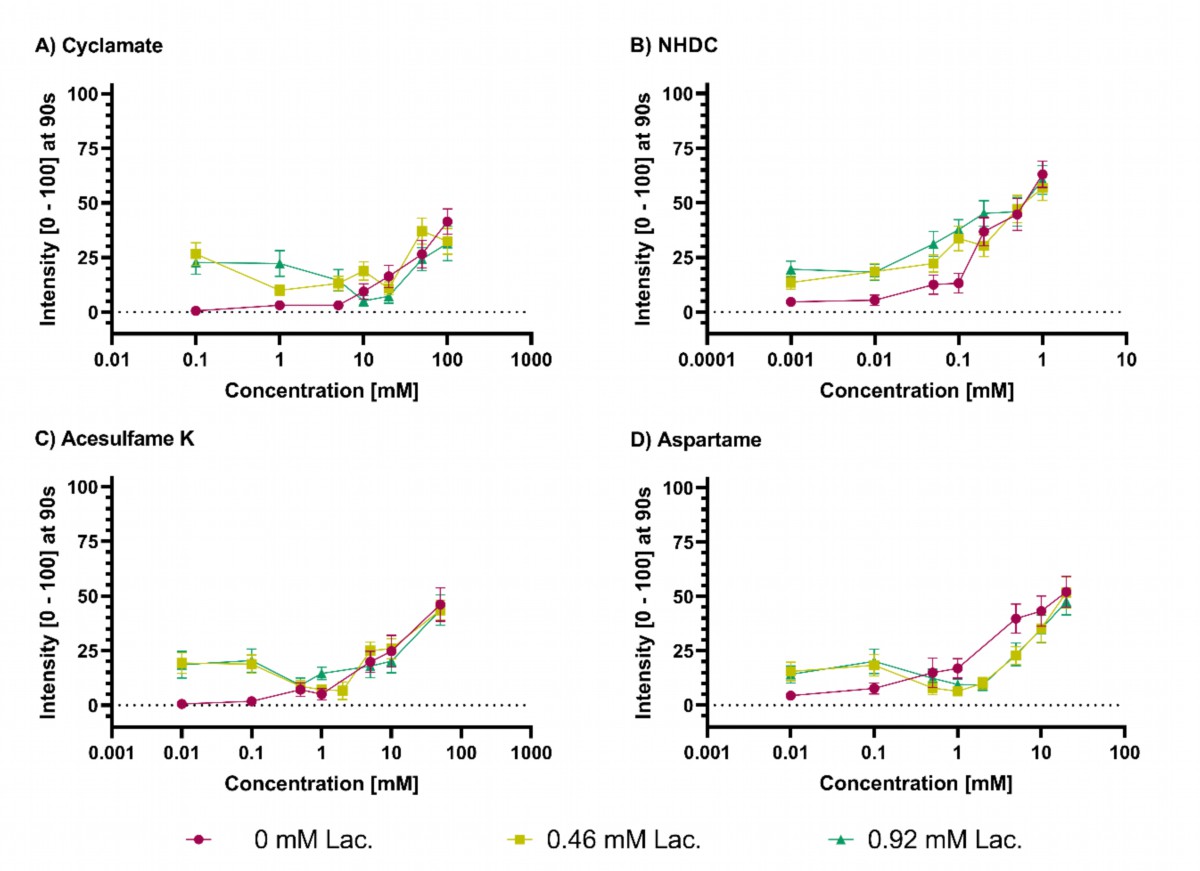


Supplemental Fig. S2: Lingering shown as intensity [0 ‑ 100] at t= 90 s of cyclamate (0.1 ‑ 100mM), NHDC (0.001 ‑ 1.0mM), acesulfame K (0.01 ‑ 50mM) and aspartame (0.01 ‑ 20mM); presented as mean ± SEM; 2 rep. with n= 18 ‑ 27 single evaluations for combination with 0 mM, 0.46 mM and 0.92 mM lactisole (Lac.).
